# Bipolar Optical Code Division Multiple Access Techniques Using a Dual Electro-Optical Modulator Implemented in Free-Space Optics Communications

**DOI:** 10.3390/s20123583

**Published:** 2020-06-24

**Authors:** Shin-Pin Tseng, Eddy Wijanto, Po-Han Lai, Hsu-Chih Cheng

**Affiliations:** 1 Department of Electronic Engineering, National United University, Miaoli 36003, Taiwan; sptseng@nuu.edu.tw; 2 Department of Electro-Optical Engineering, National Formosa University, Yunlin 632, Taiwan; d0777106@gm.nfu.edu.tw (E.W.); carl60296@gmail.com (P.-H.L.)

**Keywords:** bipolar, optical code division multiple access, electro-optical modulator, free-space optics communication

## Abstract

This study developed a bipolar optical code division multiple access (Bi-OCDMA) technique based on spectral amplitude coding for the formation and transmission of optical-polarized and coded signals over wireless optical channels. Compared with conventional Bi-OCDMA schemes, the proposed free-space optics communication system that uses a dual electro-optical modulator design improves the transmission rate. In theory, multiple access interference can be removed by using correlation subtraction schemes. The experiment results revealed that the proposed system can be employed to accurately extract codewords from an *M*-sequence and subsequently reconstruct the desired original data. Moreover, the proposed architecture can be implemented easily in simple and cost-effective designs and may be beneficial for broadening the use of Bi-OCDMA schemes in wireless optical communications.

## 1. Introduction

Future fifth-generation (5G) networks require high bandwidth, low latency, accurate synchronization, and high reliability because they use key 5G technologies—namely enhanced mobile broadband that provides a peak data rate of ≥10 Gbps, massive machine-type communications (mMTCs) that transmit data among Internet of things (IoT) devices, and ultrareliable low-latency communications—with reliability and latency in the millisecond range. These requirements present numerous challenges in communication systems. Optical communication techniques are also promising candidates for overcoming such challenges because they can provide high bandwidth and a small latency. Optical communications have a small time delay because light provides high-speed transmission that improves the propagation delay and optical fibers have low attenuation that reduces the need for repeating and processing transmission signals. Furthermore, all current IoT applications, including e-health, telemedicine, surveillance systems, autonomous vehicles, and virtual reality platforms, require high bandwidth; therefore, wireless optical communications (WOC) have received considerable research attention [1,2,3,4]. Compared with conventional wireless communication techniques, WOC schemes can substantially resist electromagnetic wave interference (EMI).

A fundamental part of a WOC system is multiplexing techniques, which entail multiple users transmitting data by using a single link. In optical communication environments, the most widely used multiplexing technique is wavelength division multiplexing (WDM). WDM is advantageous for its configuration simplicity; however, it has disadvantageous spectral efficiency. In 2019, Ahmed et al. used WDM in free-space optical (FSO) communications to improve the performance of a system with a frequency range in the visible light spectrum [1].

Time division multiplexing (TDM) is another multiplexing scheme that allows users to simultaneously access the same channel by assigning time slots to all users. Although TDM schemes have sufficient spectral efficiency, they are subject to nonlinear fiber effects. In 2014, Mahloo et al. proposed a hybrid WDM–TDM approach for passive optical networks to increase the number of users in FSO communication systems while maintaining bandwidth [2]. Hybrid WDM–TDM combines the advantages of WDM and TDM to increase the number of users and achieve long-range communication. In addition, space division multiplexing (SDM) involves the application of beam separation to simultaneously deliver different data streams. In 2019, Rommel et al. proposed SDM with multicore fibers, and they used optical beamforming to access high-capacity millimeter-wave radios [3]. In 2019, Li et al. designed a novel quadrant detector to improve WOC transmission [4].

Some studies have reported a novel multiplex technique, namely optical code division multiple access (OCDMA) [5,6,7,8,9,10,11,12,13,14,15,16,17,18,19,20,21]. OCDMA employs CDMA techniques in optical fiber communication environments. This multiplexing scheme uses an optical coding technique in which a channel assigns each user a unique codeword to prevent mutual interference in the same channel. This technique allows the simultaneous transmission of unsynchronized data from multiple users of the same channel and bandwidth [5,6]. Therefore, OCDMA has favorable antijamming properties and moderate security with high-capacity processing. Among OCDMA schemes, spectral amplitude coding (SAC) is the most effective for alleviating multiple access interference (MAI).

On the basis of optical signal demodulation, OCDMA techniques can be divided into two categories. First, an incoherent OCDMA system uses optical field intensity to encode optical signals. These systems mainly use unipolar encoding (0, 1), which has a simple system structure and cost-effective design. However, the number of codewords that can be obtained through unipolar encoding is considerably smaller than that obtained through bipolar coding. To increase the number of simultaneous users, the code length must be increased, but this increases the system cost. Second, coherent OCDMA systems use the spectral phase of light to encode signals and a matching filter to control the optical phase [7]. These systems use bipolar encoding (−1, 1). Because bipolar codes have pseudo-orthogonality, the value of a cross-correlation function between any two codewords can be approximated to 0, which results in low MAI and considerably enhances system performance. In addition, Zefreh et al. introduced a power-cubic nonlinear preprocessor for improving the coherent SAC OCDMA system performance; through numerical calculations, they demonstrated that MAI is the dominant noise in high-power scenarios [8].

Furthermore, unipolar and bipolar OCDMA techniques increase the security of communication networks [9,10].

In 2006, Chang et al. developed a spectral polarization coding approach for implementing complementary bipolar optical correlation in an incoherent bipolar OCDMA (Bi-OCDMA) network [11]. Each decoder employed several fiber Bragg gratings (FBGs) and polarization beam splitters to construct differential photodetectors. The spectral amplitude was incorporated into polarization coding as a specific address code. Their complementary bipolar spectral polarization coding scheme used Hadamard codes as optical codewords for each user, and the coded optical signals were then assigned to either a vertical or horizontal polarization state for polarization coding. Although MAI could be eliminated through correlation subtraction for differential photodetectors, their system had high complexity.

In 2007, Zeng et al. implemented a unipolar-encoding/bipolar-decoding OCDMA scheme that used an electro-optic phase modulator and two FBG arrays in system design [12]. On the transmitter side, a data sequence was used to modulate the phase of the optical carriers, and an FBG encoder array was used for wavelength mapping to an optical phase sequence. On the receiver side, an FBG decoder array was employed as a frequency discriminator to convert phase-modulated optical signals into intensity-modulated signals for optical decoding. However, the decoders used a series of FBGs, which further limited the rate of signal transmission.

In 2018, Patel et al. developed a double-weight code pattern for bipolar codes by using a reconfigurable encoder design [13]. The design increased security against eavesdroppers at the transmitting end. To reconstruct the information of a desired user, the receiver employed complementary subtraction and single photodiode detection. However, the pattern of the code used was straightforward.

In 2019, Filho et al. compared the encoding and decoding of both bipolar and unipolar sequences by using a superstructure FBG (SFBG) [14]. They evaluated SFBG performance in autocorrelation and cross-correlation. This enabled the measurement of unipolar and bipolar coding quality and the effect of multiple users on a network.

In 2020, Ghoumid et al. developed a Bi-CDMA system with a phase shift by applying an E-beam technique to a H_x_Li_1−x_NbO_3_ transmission channel by using several cascaded Bragg filters and Hadamard codes to conduct several experiments [15]. Because phase shifts must be identified on a coder spectral response, their entire structure is relatively complex. To improve system performance, some researchers have proposed an intelligently structured receiver to suppress noise effects and a semiconductor optical amplifier (SOA) to mitigate temperature variation effects on links [16,17]. In 2018, Yen et al. presented Walsh–Hadamard-code-based OCDMA techniques with moderate security for applications in WOC environments [18].

We developed a simple Bi-OCDMA FSO system by using a family of *M*-sequences and dual electro-optical modulator (EOM) schemes. In this study, the corresponding system was simplified and had the advantages of small size, cost effectiveness, and moderate security. Furthermore, compared with conventional SAC OCDMA schemes, the proposed Bi-OCDMA techniques retained the benefits of the same SAC codec design, MAI alleviation, and complementary keying to enhance overall transmission performance. However, there is a restriction in keeping the properties of two EOMs as similar as possible. Subsequently, we conducted an experiment to test the proposed scheme. The experimental results revealed that the transmission rates for each user can be improved.

The rest of this paper is organized as follows: Section 2 describes the proposed FSO communication system that uses Bi-OCDMA schemes and includes explanation of the coding theory, corresponding system design, and operation. Section 3 details the experimental setup and the results of Bi-OCDMA encoding and decoding. Section 4 describes the FSO system and provides conclusions.

## 2. Development of the Proposed FSO Bi-OCDMA System

*M*-sequences have been used to develop SAC and all-fiber loop vibration sensor systems [19,20]. A family of *M*-sequences that forms all sequences of the same length is used in Bi-OCDMA schemes. Let ***X***_1_ be a codeword from *M*-sequences as follows:

(1) $$X_{1}=\left[x_{1}\left(1\right),\;x_{1}\left(2\right),\ldots ,\;x_{1}\left(N\right)\right]$$

where *x_k_*(*i*) is the *i*th element of the *k*th codeword of the *M*-sequence and *N* is the code length of the *M*-sequence. Subsequently, the cyclic property of *M*-sequences is used to easily generate codewords of the same length *N* through an operation of ***X***_(*k*+1)_ = *T^k^**X***_1_, where *k* is the number of cyclic shifts to the right side. Consider the *M*-sequence for which *N* = 3. The codeword then assigned to each user can be explained as follows:

(1) Codeword assigned to the first user: ***X***_1_ = [101];
(2) Codeword assigned to the second user: ***X***_2_ = *T*^1^***X***_1_ = [110];
(3) Codeword assigned to the third user: ***X***_3_ = *T*^2^***X***_1_ = [011].

With polarization coding and modulation techniques, Bi-OCDMA schemes using *M*-sequence codes can be implemented as follows: The optical signal corresponding to the assigned codeword with a vertical polarization state is transmitted when the data bit of the *k*th user is 1, and that corresponding to the assigned codeword with a horizontal polarization state is transmitted when the data bit of the *k*th user is 0. Table 1 presents *M*-sequences for which *N* = 3 with a bipolar scheme. The subscripts V and H represent optical signals with vertical and horizontal polarization states, respectively. To employ *M*-sequences in the proposed Bi-OCDMA schemes, the results of a correlation with length *N* could be obtained as follows:

(2) $$R_{XX}^{\left(nm\right)}\left(j,k\right)=\sum_{i=1}^{N}x_{j}^{\left(n\right)}\left(i\right)x_{k}^{\left(m\right)}\left(i\right)=\{\;\begin{array}{ccc} \left(N+1\right)/2 & , & \;\mathrm{for}\;j=k\;\mathrm{and}\;n=m\\ \left(N+1\right)/4 & , & \;\mathrm{for}\;j\neq k\;\mathrm{and}\;n=m\\ 0 & , & \;\mathrm{otherwise}\; \end{array}$$

and

(3) $$R_{XX}^{\left(nm\right)}\left(j,\bar{k}\right)=\sum_{i=1}^{N}x_{j}^{\left(n\right)}\left(i\right)\left[1-x_{k}^{\left(m\right)}\left(i\right)\right]=\{\;\begin{array}{ccc} \left(N+1\right)/4 & , & \;\mathrm{for}\;j\neq k\;\mathrm{and}\;n=m\\ 0 & , & \;\mathrm{otherwise}\; \end{array}$$

where *n* and *m* represent the optical codewords with individual horizontal and vertical polarization states, respectively. Theoretically, similar to SAC techniques, the following equations can be employed to prevent the influence of MAI.

(4) $$\begin{array}{lll} \left[R_{XX}^{\left(nV\right)}\left(j,k\right)+R_{XX}^{\left(nH\right)}\left(j,\bar{k}\right)\right] & - & \left[R_{XX}^{\left(nV\right)}\left(j,\bar{k}\right)+R_{XX}^{\left(nH\right)}\left(j,k\right)\right]\\ & & \;\;=\{\;\begin{array}{ccc} \left(N+1\right)/2 & , & \;\mathrm{for}\;j=k\;\mathrm{and}\;n=V\\ -\left(N+1\right)/2 & , & \;\mathrm{for}\;j=k\;\mathrm{and}\;n=H\\ 0 & , & \;\mathrm{otherwise}\; \end{array} \end{array}$$

On the basis of these deductions, the corresponding FSO system using Bi-OCDMA schemes can be developed.

**Figure 1:**
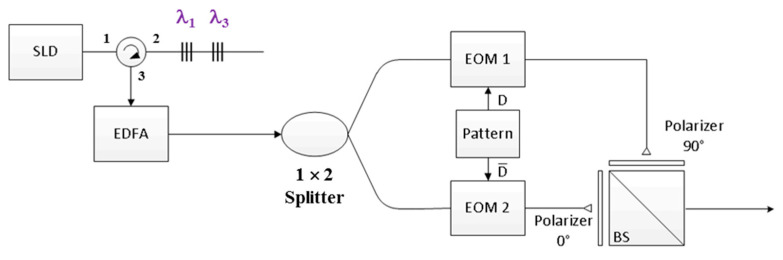
Proposed bipolar optical code division multiple access encoder.

Figure 1 illustrates the design of the proposed FBG encoder with the *M*-sequence for which *N* = 3. The proposed encoder comprises a superluminescent diode (SLD) light source, an optical circulator, a series of FBGs, a 1 × 2 optical splitter, a pattern generator, two polarizers (0° and 90°), a beam splitter (BS), and two EOMs for bipolar coding. First, an SLD output is inserted into Port 1 of the optical circulator and then used as an input for the series of FBGs through Port 2 of the optical circulator. Subsequently, the series of FBGs is employed to reflect specific wavelengths according to the codeword assigned using the *M*-sequence. For example, when *N* = 3, the FBG resonance wavelengths are λ_1_ and λ_3_, corresponding to codeword ***X***_1_ = [1 0 1] for the first user.

Subsequently, the reflected optical signals are entered as an input into Port 2 of the optical circulator, which then provides output from Port 3. Subsequently, these optical signals are coupled into the 1 × 2 optical splitter through an erbium-doped fiber amplifier (EDFA) to compensate for the attenuation of devices in the encoder. The amplified signals are distributed in a parallel manner in the two EOMs for dual EOM modulation. The output signals of the two EOMs are then determined according to the normal (D) and complementary ($\bar{D}$) outputs of the pattern generator, where D is the data bit of the user, represented as “0” or “1”. No optical signal appears at the output port of EOM 2 for user data bits of “1”. By contrast, no output signal appears at the output port of EOM 1 for user data bits of “0”. The outputs of EOMs 1 and 2 are entered as inputs into the vertical and horizontal polarizers, respectively, for polarization coding and are then combined through the BS. For example, if the data bit of user 1 is “1,” the BS output corresponds to [1 0 1]_V_ and [0 0 0]_H_; however, if the data bit of user 1 is “0,” the BS output corresponds to [0 0 0]_V_ and [1 0 1]_H_. Finally, the output of each encoder is coupled into a fiber collimator and transmitted via a wireless optical channel.

**Figure 2:**
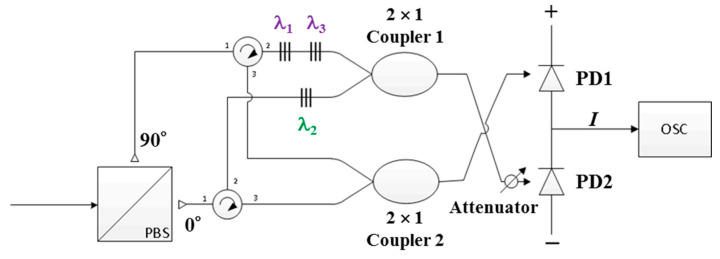
Structure of the proposed bipolar optical code division multiple access decoder.

During the receiving process, the wireless optical signal is received through a fiber collimator and then distributed to the input port of each decoder. Figure 2 illustrates the structure of the proposed FBG-based Bi-OCDMA decoder, which contains a polarization beam splitter (PBS), two optical circulators, two series of FBGs, two 2 × 1 optical couplers, an attenuator, and a balanced photodetector (BPD) to subtract upper and lower signals and mitigate potential MAI.

First, the optical signals received from the collimator output are depolarized through the PBS and then used as input for the first ports of two circulators. The received optical signals with vertical and horizontal polarization components appear in the upper arm and lower branch paths, respectively. The two ports of the two optical circulators are connected to the two series of FBGs, which correspond to the normal (***X***) and complementary ($\bar{X}$) codewords. For example, when *N* = 3, the series of upper FBGs reflects the central wavelengths of λ_1_ and λ_3_ that correspond to the normal codeword ***X***_1_ = [1 0 1] in FBG Decoder 1. Similarly, the second series of FBGs reflects the central wavelength of λ_2_, which corresponds to the complementary codeword ${\bar{X}}_{1}$ = [0 1 0] in FBG Decoder 1. The output signals of the two series of FBGs are coupled into the upper 2 × 1 optical coupler. The lower optical coupler is used to collect optical signals from Port 3 of each of two optical circulators. The output signal received from the upper coupler passes through the attenuator and arrives at the second input port of the BPD. The purpose of this process is to alleviate the influence of unwanted spectral outputs caused by imperfect reflections in the FBG decoder. The output signal of the lower coupler is used as an input to the first input port of the BPD. On the basis of Equations (2) and (3), we developed two models for the outputs of the upper and lower couplers of FBG Decoder 1, namely ***F***_11_ and ***F***_21_, when the codeword of X*_j_* is received, expressed as

(5) $$F_{11}=\left[R_{XX}^{\left(nH\right)}\left(j,1\right)+R_{XX}^{\left(nV\right)}\left(j,\bar{1}\right)\right]=\{\;\begin{array}{ll} 0, & \;\mathrm{for}\;j=1\;\mathrm{and}\;n=V\\ 2, & \;\mathrm{for}\;j=1\;\mathrm{and}\;n=H\\ 1, & \;\mathrm{for}\;j=2,3 \end{array}$$

and

(6) $$F_{21}=\left[R_{XX}^{\left(nV\right)}\left(j,1\right)+R_{XX}^{\left(nH\right)}\left(j,\bar{1}\right)\right]=\{\;\begin{array}{ll} 2, & \;\mathrm{for}\;j=1\;\mathrm{and}\;n=V\\ 0, & \;\mathrm{for}\;j=1\;\mathrm{and}\;n=H\\ 1, & \;\mathrm{for}\;j=2,3 \end{array}$$

where $R_{XX}^{\left(nV\right)}\left(j,\bar{1}\right)$ and $R_{XX}^{\left(nH\right)}\left(j,1\right)$ are the optical signals at the upper and lower input ports of the upper coupler, respectively. The expressions $R_{XX}^{\left(nV\right)}\left(j,1\right)$ and $R_{XX}^{\left(nH\right)}\left(j,\bar{1}\right)$ are the optical signals at the upper and lower input ports of the lower coupler, respectively. These optical signals arrive at the input ports of the BPD for correlation subtraction and MAI removal according to the results of Equation (4) in Section 2 and are then converted into electrical signals. Therefore, the output of the BPD can be converted into model (***F***), as follows, when the codeword of X*_j_* is received:

(7) $$F=F_{21}-F_{11}=\{\;\begin{array}{cc} 2, & \;\mathrm{for}\;j=1\;\mathrm{and}\;n=V\\ -2, & \;\mathrm{for}\;j=1\;\mathrm{and}\;n=H\\ 0, & \;\mathrm{otherwise}\; \end{array}$$

Finally, a decision current (*I*) is used to determine the data bit of the desired user from the wireless optical channel.

## 3. Experimental Setup and Results

**Figure 3:**
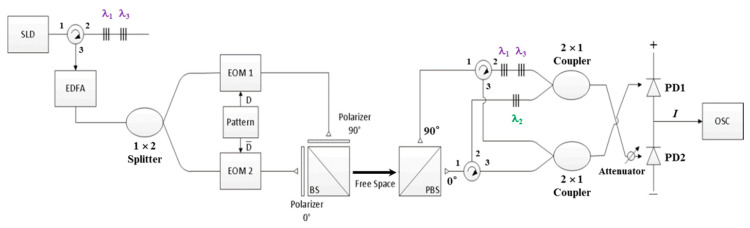
Proposed free-space optical communication system using bipolar optical code division multiple access and dual electro-optical modulator schemes.

On the basis of the structure illustrated in Figure 3 with *N* = 3, the feasibility of the proposed FSO communication system was verified through several experiments by using a model with the following specifications: (1) for the light source, the NXTAR SLD-2000 was adopted. (2) Couplers (Fiber Optic Communications, Inc., Hsinchu, Taiwan) were used as 1 × 2 splitters and 2 × 1 couplers. (3) A Pirelli 10-Gbps integrated optic intensity modulator—which used two EOMs—was used to modulate the signal of the pattern output. (4) An Agilent 81130A pulse pattern generator was used to generate desired patterns for transmission. (5) A left-handed plastic circular polarizer (CP42HE) 12.5 mm in diameter—which used two polarizers (0° and 90°)—was used to assign the light signal to the specific polarization state. (6) A 25-mm nonpolarizing cube BS with a wavelength range of 1100–1620 nm was used to combine optical signals from different paths. (7) A 5-mm VIS polarizing cube BS was used to split two polarization states (0° and 90°) from the input optical signal. (8) A single-mode circulator (1550 nm and 500 mW; FCIR-1550-3-3-A-0-1-2-1-2) was used as the optical circulator. (9) The attenuator range was adjusted from 0 to −30 dB to alleviate the influences of noise and the lower branch signal in the front of PD2. (10) The BPD (Model-1817, New Focus Inc., USA) was used for optical signal subtraction and the conversion of the results into electrical signals. (11) A Tektronix oscilloscope (OSC, model TDS2102B) was used to monitor BPD outputs. (12) A limited power supply of 15 V was provided to the BPD. (13) An Anritsu MS9710C optical spectrum analyzer was employed to assess the accuracy of the spectral output acquired from a codec.

Assume that the first user is the desired user. First, the FBG resonance wavelengths used for the FBG codec are 1543, 1546, and 1549 nm for λ_1_, λ_2_, and λ_3_, respectively. Therefore, user 1 is assigned the codeword ***X***_1_ = [λ_1_ 0 λ_3_].

**Figure 4:**
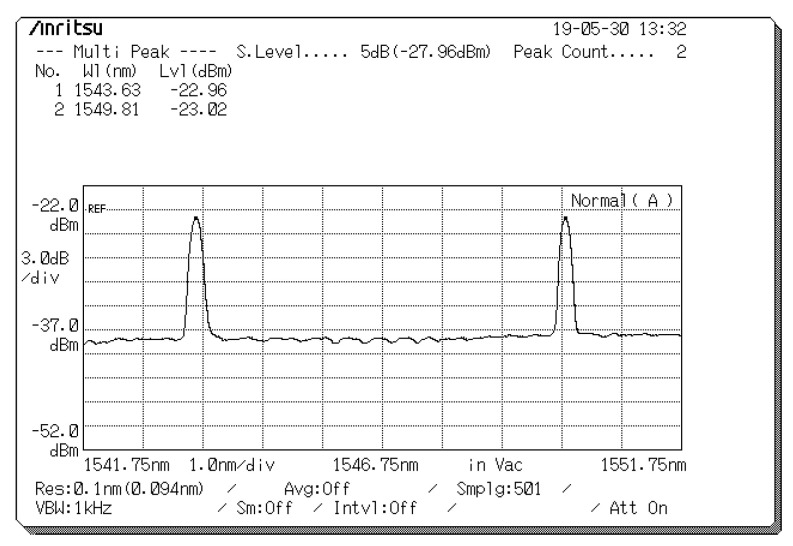
Reflected spectra (λ_1_, λ_3_) used for user 1.

Figure 4 presents the measured reflected spectra (λ_1_, λ_3_) for user 1 with corresponding central wavelengths of 1543 and 1549 nm and light intensities of −22.96 and −23.02 dBm, which appeared at the circulator port in Encoder 1. After the reflected spectra passed through the EDFA and 1 × 2 splitter, they were modulated by EOMs 1 and 2 according to the normal (D) and complementary ($\bar{D}$) outputs of the pattern generator, and the spectra were then entered as inputs that were parallel to the input ports of the two polarizers (90° and 0°) to determine suitable polarization states.

**Figure 5:**
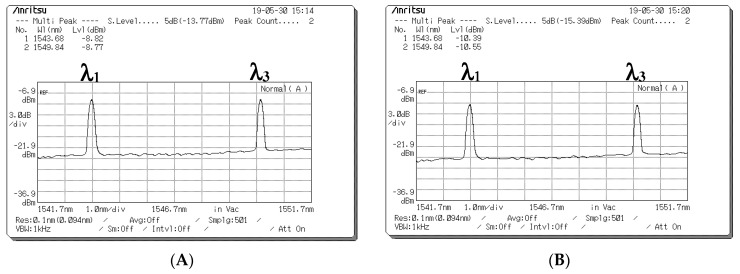
Output spectrum acquired at the output ports of the two electro-optical modulators (EOMs) for different data bits. (**A**) Optical spectra of EOM 1 for a data bit of “1”. (**B**) Optical spectra of EOM 2 for a data bit of “0”.

**Figure 6:**
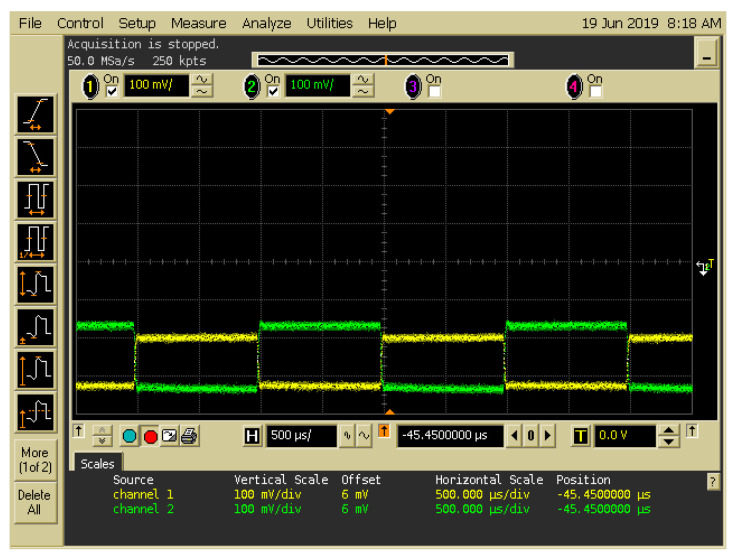
Input signals of the two electro-optical modulators (EOMs) when the signals of Channels 1 and 2 were entered as inputs into EOM 1 and EOM 2, respectively.

Figure 5 presents the spectra obtained at the output ports of the two EOMs operating with different data bits. Figure 5A presents the output spectrum obtained at the output port of EOM 1 when the data bit (D) of user 1 is “1”. The central wavelengths of λ_1_ and λ_3_ are 1543 and 1549 nm, respectively, and the corresponding light intensities are −8.82 and −8.77 dBm, respectively. Figure 5B presents the output spectrum obtained at the output port of EOM 2 when the data bit (D) of user 1 is “0”. The central wavelengths of λ_1_ and λ_3_ are 1543 and 1549 nm, respectively, and the corresponding light intensities are −10.39 and −10.55 dBm, respectively. Figure 6 indicates that a signal frequency of 500 Hz was acquired from pattern generation and entered as an input into EOMs 1 and 2 of Encoder 1, where the input signals of the two EOMs complemented each other. The output signals of the EOM 1 and EOM 2 were assigned polarization states of 0° and 90°, respectively, and they were then combined into a free-space channel passing through the BS and collimator.

**Figure 7:**
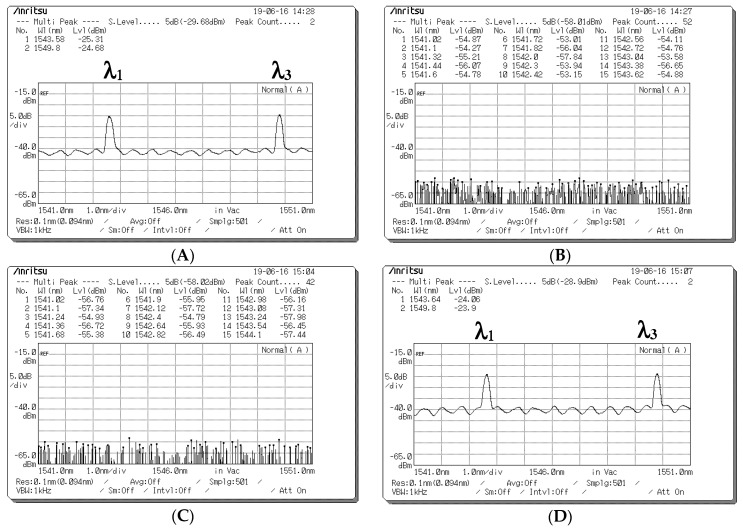
Optical spectrum acquired at the output ports of the polarization beam splitter (PBS) in Decoder 1 when different data bits (D) are sent from Encoder 1. Depolarized spectra acquired at (**A**) Output Port 1 and (**B**) Output Port 2 of the PBS when a data bit of “1” is sent. Depolarized spectra acquired at (**C**) Output Port 1 and (**D**) Output Port 2 of the PBS when a data bit of “0” is sent.

Figure 7 presents the depolarized spectra obtained at the output ports of the PBS in Decoder 1 when the encoded signal with different data bits was received from Encoder 1. Figure 7A,B presents the depolarized spectra acquired at the output ports of the PBS in Decoder 1 when a data bit (D) of “1” is transmitted. In the depolarized spectra (λ_1_, λ_3_) with the 90°polarization state at the first output port of the PBS, the corresponding central wavelengths were 1543 and 1549 nm, and the light intensities were −25.31 and −24.68 dBm, respectively (Figure 7A). Figure 7B indicates that no signal appeared at the second output port of the PBS when the data bit (D) of user 1 was “1”. Figure 7C,D presents the depolarized spectra acquired at the output ports of the PBS in Decoder 1 when a data bit (**D**) of “0” is transmitted. Figure 7C indicates that no signal appeared at the first output port of the PBS when the data bit (D) of user 1 was “0”. In the depolarized spectra (λ_1_, λ_3_) with the 0° polarization state at the second output port of the PBS, the corresponding central wavelengths were 1543 and 1549 nm, and the light intensities were −24.06 and −23.9 dBm, respectively (Figure 7D). Therefore, in the experiment, the degree of light intensity demonstrated an approximate 13.35–16.5-dB loss from the EOM output in Encoder 1 to the PBS output in Decoder 1 through the wireless optical channel.

**Figure 8:**
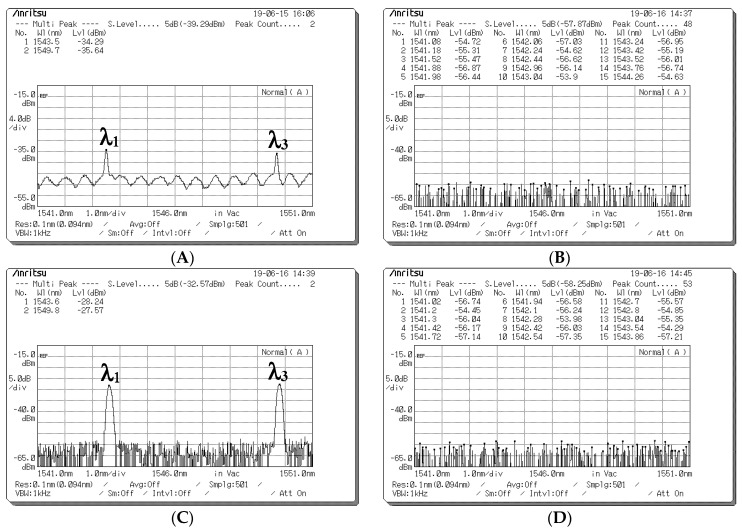
Optical spectrum before the optical signals entered upper and lower couplers when a data bit (D) of “1” was sent from Encoder 1. (**A**) Optical spectra obtained at Port 1 of the upper coupler when the optical signals passed through fiber Bragg grating (FBG) Decoder 1. (**B**) Optical spectra obtained at Port 2 of the upper coupler when the optical signals passed through the 0° component path. (**C**) Optical spectra obtained at Port 1 of the lower coupler when the optical signals passed through FBG Decoder 1. (**D**) Optical spectra obtained at Port 2 of the lower coupler when the optical signals passed through the 0° component path.

Figure 8 presents the measured spectra before the optical signals entered the upper and lower couplers when a data bit (D) of “1” is sent from Encoder 1. Figure 8A presents the spectra measured using the upper optical circulator and FBG Decoder 1 before input was entered in Port 1 of the upper coupler when the a data bit (D) of user 1 was “1”. The corresponding central wavelengths were 1543 and 1549 nm, and the light intensities were −34.29 and −35.64 dBm, respectively. Figure 8B indicates that no signal appeared at Port 2 of the upper coupler for user 1’s data bit (D) of “1” when the spectra passed through the horizontal (0° component) path; the corresponding central wavelengths were 1543, 1546, and 1549 nm, and all light intensities were less than −53 dBm. Figure 8C presents the decoded spectra that passed through the upper optical circulator and FBG Decoder 1 before it was entered as an input into Port 1 of the lower coupler when the data bit (D) of user 1 was “1”. The corresponding central wavelengths were 1543 and 1549 nm, and the light intensities were −28.24 and −27.57 dBm, respectively. Figure 8D indicates that no signal appeared at Port 2 of the lower coupler for user 1’s data bit (D) of “1” when the spectra passed through the horizontal (0° component) path; the corresponding central wavelengths were 1543, 1546, and 1549 nm, and all light intensities were less than −53 dBm. Some depolarized spectral outputs were generated because of the imperfect reflection obtained from FBG Decoder 1 (Figure 8A).

**Figure 9:**
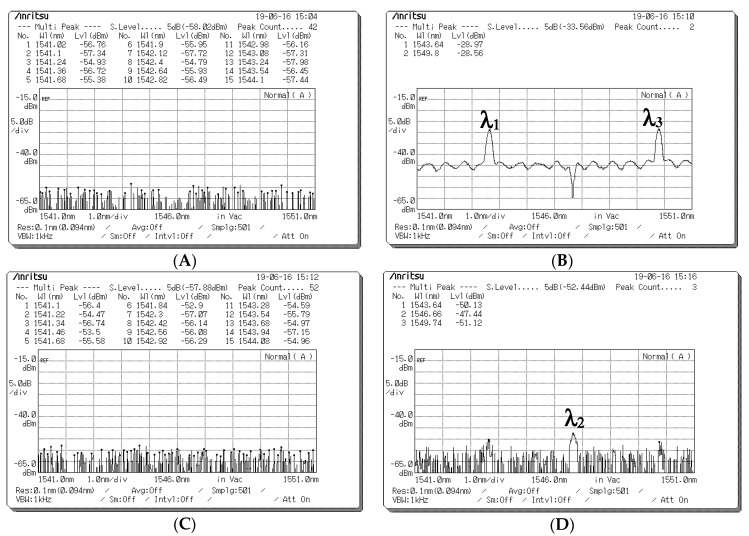
Output spectrum before optical signals entered the upper and lower couplers when a data bit (D) of “0” was sent from Encoder 1. (**A**) Optical spectra acquired at Port 1 of the upper coupler when the signal passed through the 90° component path. (**B**) Optical spectra acquired at Port 2 of the upper coupler when the signal passed through fiber Bragg grating (FBG) Complement Decoder 1. (**C**) Optical spectra acquired at Port 1 of the lower coupler when the signal passed through the 90° component path. (**D**) Optical spectra acquired at Port 2 of the lower coupler when the signal passed through FBG Complement Decoder 1.

Figure 9 presents the spectra measured before the optical signals entered the upper and lower couplers when a data bit (D) of “0” was sent from Encoder 1. Figure 9A indicates that no signal appeared at Port 1 of the upper coupler for user 1’s data bit (D) of “0” when the spectra passed through the vertical (90° component) path; the corresponding central wavelengths were 1543, 1546, and 1549 nm, and all light intensities were less than −53 dBm. Figure 9B presents the spectra decoded using the lower optical circulator and FBG Complement Decoder 1 before they were input into Port 2 of the upper coupler for user 1’s data bit (D) of “0”. The corresponding central wavelengths were 1543 and 1549 nm, and the light intensities were −28.97 and −28.56 dBm, respectively. Figure 9C indicates that no signal appeared at Port 1 of the lower coupler for user 1’s data bit (D) of “0” when the spectra passed through the vertical (90° component) path; the corresponding central wavelengths were 1543, 1546, and 1549 nm, and all light intensities were less than −53 dBm. Figure 9D presents the optical spectra obtained through the lower optical circulator and FBG Complement Decoder 1 before they were input into Port 2 of the lower coupler for user 1’s data bit (D) of “0”. The corresponding central wavelengths were 1543, 1546, and 1549 nm, and all light intensities were less than −47 dBm. Some unwanted spectral outputs were produced because of the imperfect upper coupler connection and lower circulator (Figure 9D). However, the decoded output was unaffected by leakage intensities (Figure 9D).

**Figure 10:**
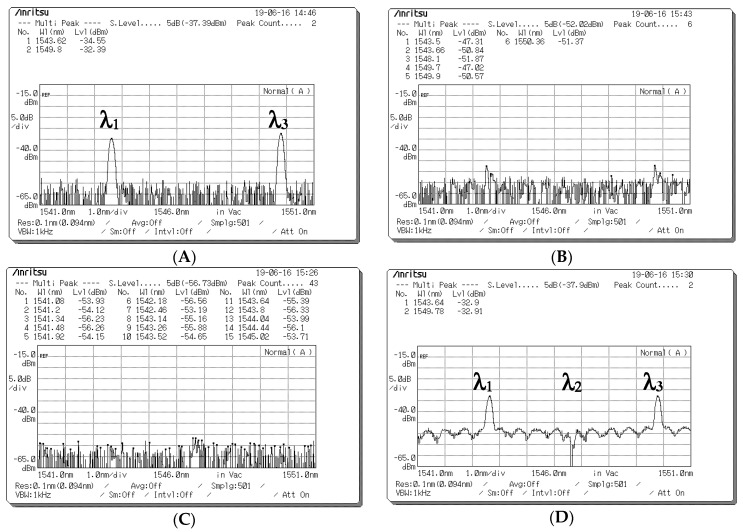
Decoded spectra appearing at the output ports of the upper and lower couplers when different data bits (D) of user 1 were sent. Optical spectra obtained at the output port of the (**A**) upper coupler and (**B**) lower coupler for a data bit (D) of “1” for user 1. Optical spectra obtained at the output port of the (**C**) upper coupler and (**D**) lower coupler for a data bit (D) of “0” for user 1.

Figure 10 presents the output spectrum obtained at the output ports of the upper and lower couplers in Decoder 1 when the different data bits (D) of user 1 were sent. Figure 10A,B presents the optical spectra appearing at the output ports of the upper and lower couplers in Decoder 1, respectively, and Figure 10C,D presents those appearing for data bits (D) of “1” and “0” entered as inputs into Encoder 1. Subsequently, the BPD converted the decoded spectra corresponding to its input ports into electrical signals.

**Figure 11:**
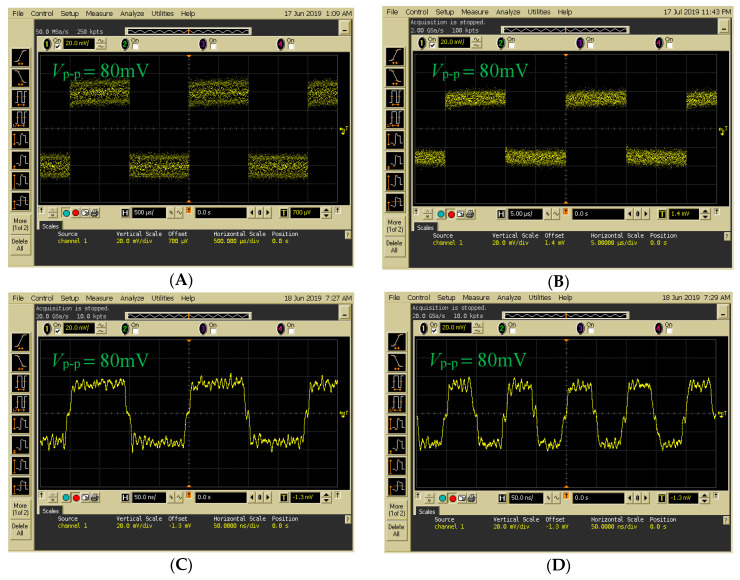
Decoding results for Decoder 1 acquired by the balanced photodetector at signal frequencies of (**A**) 500 Hz, (**B**) 50 kHz, (**C**) 5 MHz, and (**D**) 10 MHz input to Encoder 1.

Figure 11 presents the decoding results of changing frequencies when data were transmitted from Encoder 1. A digital OSC was used to access the transmitted signal from Encoder 1. In Figure 11A–D, input frequencies of 0.5, 50, 5000, and 10,000 kHz were used as inputs for FBG Encoder 1. Compared with previous systems [21], the novel FSO communication system with the proposed Bi-OCDMA scheme was implemented successfully and further enhanced the overall transmission rate.

## 4. Conclusions

In this study, the use of Bi-OCDMA with a dual EOM scheme implemented in WOC environments was proposed and successfully demonstrated at normal atmospheric temperatures. FBGs were employed as primary devices for developing the codec. The measurement results of the signal transmission rate revealed that switching limitations of previous systems using an optical switch [21] can be improved through use of the proposed design with a dual EOM structure.

The proposed Bi-OCDMA method is based on original SAC OCDMA techniques, which theoretically alleviate the MAI effect and reduce crosstalk from other FBG encoders. When deployed, the proposed FSO system exhibited excellent properties in terms of its light weight, cost effectiveness, moderate security, and EMI resistance. These properties may further enhance the overall transmission rates of WOC applications in the near future.

Future work can apply the proposed Bi-OCDMA technique to multiuser and long-distance WOC scenarios that involve MAI mitigation and performance measurement by using parameters such as the bit error rate, Q-factor, and eye diagrams.

## Figures and Tables

**Table 1:** *M*-sequence for which *N* = 3 with a bipolar scheme.

	Codeword X	Data Bit (D)	Transmitting Optical Signal
			λ_1_ λ_2_ λ_3_
User 1	1 0 1	0	[1 0 1]_H_
		1	[1 0 1]_V_
User 2	1 1 0	0	[1 1 0]_H_
		1	[1 1 0]_V_
User 3	0 1 1	0	[0 1 1]_H_
		1	[0 1 1]_V_
